# Date Palm as Source of Nutraceuticals for Health Promotion: a Review

**DOI:** 10.1007/s13668-022-00437-w

**Published:** 2022-09-20

**Authors:** Sahar Y. Al-Okbi

**Affiliations:** Nutrition and Food Sciences Department, National Research Centre, Cairo, Egypt

**Keywords:** Date palm, Bioactive ingredients, Nutrients, Health benefits

## Abstract

***Purpose of Review*:**

Chronic diseases are problematic to health professional specially when using drugs throughout the course of life with un-tolerated side effects. Returning to nature through using nutraceuticals might have both protective and therapeutic effects. Date palm was claimed to be a good source of such nutraceuticals or functional food ingredients. The purpose of the present review was to spot light on the different phytochemicals, phytonutrients, and remedial effects of date palm (*Phoenix dactylifera* L.) in a goal to be utilized in form of nutraceuticals. The possible mechanisms of action of the remedial effects were among the aim of the study.

***Recent Findings*:**

A protein hydrolyzate prepared from date seed could prevent DNA mutation and susceptibility to cancer. In addition to cancer prevention, date palm fruit improved the treatment outcome of cancer pediatric patients and possesses anti-angiogenic activity as one of the important anticancer mechanisms of action. On the other hand, date seed extracts was recently reported to protect from ulcerative colitis. It seems that all the aforementioned remedial effect might be ascribed to immunoregulatory effect of date palm. These findings proposed that date palm is beneficial for health.

***Summary*:**

Date palm fruit is a rich source of vitamins, minerals, dietary fibers, energy, and easily digestible and absorbable sugars that instantaneously replenish and revitalize the body specially after fasting condition. Mineral contents in date fruits include potassium, phosphorus, magnesium, and calcium. Diverse health claims were reported to belong to various parts of the tree including the edible part of fruits, the seeds, the leaves, spathe (an envelope-like structure that encloses male and female date palm flowers), and pollen grains due to the presence of different bioactive constituents. The main phytochemicals and phytonutrients reported in date palms are phenolic compounds, carotenoids, sterols, anthocyanins, and others. In folk medicine, date palm fruits are used for enhancing immunity and treating gastrointestinal tract disorders, edema, bronchitis, wound, cancer, as well as infectious diseases**.** However, the exact health benefits and remedial effects of date palm were not fully and deeply investigated. The present review focused on the bioactive constituents and the reported health benefits of date palm and proposed mechanism of action.

## Introduction

Returning to natural remedy represented by medicinal herbs is now preferred by patients compared to chemical synthetic drugs due to the different side effects encountered during drug administration. As an example in patient with osteoarthritis, ginger had not only efficacy in pain improvement identical to diclofenac 100 (the non-steroidal anti-inflammatory synthetic drugs) but also had no side effects [1]. The side effects of diclofenac as non-steroidal anti-inflammatory drug are summarized later on (under the “Anti-inflammatory and antinociceptive activity of date palm” section**)**. However, not all medicinal herbs have been approved for their efficacy. Also, medicinal herbs with remedial effect (pros) might also have some toxic effects (con), and patients must be caution to only use the medicinal herbs approved by the FDA. In addition, consulting the physician is very important when using such herbs with synthetic drugs to avoid unwanted interactions. More recently nutraceuticals and functional foods are a new developing era as one category of natural therapeutic and protective agents that are certainly safer than medicinal herbs. This claim might be ascribed to that nutraceuticals are extracted from plant foods that are normally and continually consumed by human certainly without any negative effect on health because the body is accustomed on their consumption [2], while most herbs (medicinal plants) are not consumed by human compared to food; therefore, medicinal herbs are expected to have some side effects compared to nutraceuticals.

Nutraceuticals are defined as bioactive constituents purified from food, either botanical or animal food sources, and could protect or treat one or more chronic diseases and sold in pharmaceutical form. Functional foods are food rich in bioactive constituents that could protect from chronic diseases and consumed in form of food products. Both functional foods and nutraceuticals might be used as protective agents from diseases or as complementary to drug that permit reduction of drug dose with consequent reduction of its adverse effect [3]. Date palm (DP) could be regarded as source of nutraceuticals for protection and management of chronic diseases and as complementary or alternative medicines.

Vitamins, minerals, and energy are present in high amounts in the fruit of DP tree *Phoenix dactylifera* L. (*P. dactylifera*) together with highly digestible and absorbable sugars that help rapid replenish of the body especially in fasting state. The different parts of the DP tree including the fleshy pericarp, the seeds (also called pits, pips, kernels, and stones), the leaves, spathe, and pollen grains possess variable health benefits that attributed to the presence of bioactive components. Such bioactive components are represented by polyphenols, selenoproteins, carotenoids, tocopherols, sterols, essential oil, and others. Claimed health benefits in folk medicine include the regulation of immunity, and treatment of bronchitis, edema, and wound as well as cancer prevention [4, 5]. Therefore, the consumption of the fruit on regular manner could afford protection from chronic diseases, gastrointestinal disorders, and resistance to infectious diseases. The DP seeds that constitute 6–12% of the whole date fruit were also reported to possess different health benefits [4] such as antioxidant effect, renoprotective activity, recovering of spermatotoxicity, protection from ulcerative colitis, and immune-regulation as could be explained later on.

Different varieties of DP are cultivated in Arab countries, North Africa, and different Gulf countries [6, 7] and consumed as major source of nutrients and food staple in such countries. In North Africa, DP varieties include Deglet Nour, Allig and Khalti are present in Tunisia, while Zaghlool, El-Barhy, Aisha, and Samani are present in Egypt; meanwhile, bekraray variety is native to Libya. In Gulf region, Ajwa, Berni, Halaoua, Nebtat Ali, Sogaai, and Sukkari are cultivated in Saudi Arabia. Khalas, Lulu, Shikat alkahalas, Sokkery, Bomaan, Sagay, Shishi, Maghool, and others are present in United Arab Emirates. Other regions than Arab countries also have different date palm varieties. The different varieties are expected to contain variable quantities of bioactive constituents with consequent diverse health benefits.

Although DP fruits are consumed as food worldwide, so far, the exact health benefits were not fully investigated. In addition; the health professionals are not aware by all such precious therapeutic uses of this plant that could relief many of the pains and diseases of human kind. The aim of the present review was to spot light on the different phytochemicals, phytonutrients, and the remedial effect of DP in a goal to be utilized in form of nutraceuticals or functional food for combating chronic diseases. The possible mechanisms of action of the remedial effects were among the aim of the review.

## Methods

Searching literature was implemented through applying the keywords represented by date palm, fruit, seeds, different parts, health benefits, bioactive ingredients, functional ingredients, and nutraceuticals. Different database were used including PubMed, Medline, Google Scholar, ScienceDirect, and ResearchGate, in addition to researches carried out by our team. The present review was keen to focus on both experimental studies on animals and clinical trials on human.

## Nutrients’ Composition of Date Palm

Nutrients’ composition in date fruits differs according to the variety, the cultivated land, and the geographical region. Dates edible parts were demonstrated to contain 72.8–79.1% sugar represented by mainly fructose, glucose, and sucrose and 14.4–18.4% dietary fibers in addition of low percentage of protein, ash, and fat as assessed in Deglet Nour and Allig varieties in Tamr stage (full ripeness) from Tunisia [8]. Date fruit varieties from Saudi Arabia (Ajwa, Berni, Halaoua, Nebtat Ali, Sogaai, and Sukkari) were reported to contain 2.1 ± 0.2–3.1 ± 0.1% protein, 0.08 ± 0.01–7.33 ± 0.33% fat, 1.9 ± 0.3–3.4 ± 0.2% fibers, 59.6–76.8% carbohydrates, and 5.23 ± 0.02–6.20 ± 0.09% ash [9]. Dates were demonstrated to contain phosphorus, iron, potassium, and calcium [10]. Consumption of 100 g of date palm on daily basis was reported to provide half the daily dietary recommended requirements from iron, zinc, and vitamin A precursor [11]. Different varieties of dates cultivated in United Arab Emirates (18 varieties) were shown to contain sodium, potassium, magnesium, copper, manganese, and chromium in addition to the aforementioned minerals but with different levels [12]. Water-soluble vitamins were shown to present in a variable quantity in the fresh mature fruits of different varieties from United Arab Emirates. Thiamine was approximately 12, 8, and 1 µg/100 g in Khalasah, Shishi, and Muzati, respectively. Nicotinamide was around 5, 6, 35, 34, and 45 µg/100 g in Khalasah, Muzati, Barhee, Zart, and Zardi, respectively. Pantothenic acid was found to be nearly 7 µg/100 g in Shishi and 1 µg/100 g in Muzati, while pyridoxine was present as around 0.1, 0.2, 0.3, 0.15, and 0.15 µg/100 g in Khalasah, Shishi, Muzati, Zart, and Zardai, respectively. Folic acid was present as about 0.2 and 0.5 µg/100 g in Khalasah and Zardai; meanwhile, cyanocobalamin was 0.6, 0.7, 0.15, 0.2, and 0.2 µg/100 g in Khalasah, Shishi, Muzati, Zart, and Zardai, respectively [13].

Carotenoids are considered phytochemicals; however, from which β-carotene is vitamin A precursor which was present as 1342 µg/Kg in Khalas DP variety in the lipid fraction [14]. Date fruit contains moderate amount of α-tocopherol acetate in the lipid portion [15].

DP seeds are reported to contain high level of minerals and vitamins. The seeds contain fibers as 73.83–82.37%, protein as 5.23–7.02%, oil as 4.88–12.737%, moisture as 6.88–33.61%, ash as 1–2%, and total sugars as 1.21 to 8.12% [16–18].

It was reported that the seeds from 14 varieties of date contain 5.4–9.3% oil [19]. The lipid content of date palm fruits ranges from 0.12 to 0.72% [16, 17, 20]. Major fatty acids in date fruit are palmitic, oleic, and linoleic in addition of appreciable amount of linolenic [17]. In date seeds, the major fatty acids were lauric, myristic, palmitic, and oleic.

Vitamin E (tocopherols and tocotrienols) is fat-soluble vitamin present in the lipid portion of DP seed. Tunisian date seed was reported to contain α-tocotrienol (34.01 mg/100 g), γ-tocotrienol (4.63 mg/100 g), and γ-tocopherol (10.3 mg/100 g) [21]. In Khalas variety, α-tocopherol was present as 24.3 mg/100 g in seed oil and as 19.8 mg/100 g in palm kernel oil according to Al Juhaimi et al.’s study [22]. Alpha-tocopherol was found to be 24.97–38.85% in Deglet Nour and Allig date seeds [23] which looks extremely high compared to Al Juhaimi study.

The macronutrients, minerals, and fatty acids in both date fruit edible part and seeds are present in Tables 1, 2 and 3, respectively.

**Table 1:** Macronutrients in date fruit edible part and seed (g/100 g) on dry basis

	Date fruit (edible part)			Date seed			
	1	2	3	4	5	6	7
Total sugars	72.8 ± 027–79.1 ± 0.8 [8]	63.38 ± 0.34	71.2 ± 0.1–81.4 ± 0.04	5.443 ± 0.054–5.653 ± 0.187	8.12 ± 0.19	-	1.21 ± 0.01–3.81 ± 0.01
Total dietary fibers	14.4 ± 1.12–18.4% ± 0.45 [8]	-	-	-	-		73.83 ± 0.33–82.37 ± 0.01
Protein	2.1 ± 0.1–3.02 ± 0.13 [8]	3.86 ± 0.08	1.72 ± 0.05–4.73 ± 0.01	-	5.31 ± 0.09	-	5.23 ± 0.02– 7.02 ± 0.01
Lipids	0.227 ± 0.005–0.283 ± 0.014 [16]	0.26 ± 0.01	0.12 ± 0.003–0.72 ± 0.002	10.13 ± 0.033–12.737 ± 0.110	8.33 ± 0.11	5.4–9.3%	4.88 ± 0.02–7.81 ± 0.04
Ash	2.5 ± 0.04–2.52 ± 0.01 [8]	-	1.68 ± 0.01–3.94 ± 0.02	1.107 ± 0.005–1.17 ± 0.056	-	-	0.92 ± 0.01–1.35 ± 0.03
Moisture	-	39.25 ± 0.44	10.5 ± 0.1–29.5 ± 0.2	-	6.88 ± 0.03	-	16.06 ± 0.03–33.61 ± 0.06

Values (mean ± S.D)

*1:* Two varieties (Deglet Nour and Allig), Tunisia [8, 16], *2:* Khalti, Tunisia [17], *3:* 10 varieties from Saudi Arabia [20], *4:* Two varieties (Deglet Nour and Allig), Tunisia [16], *5:* Khalti, Tunisia [17], *6:* 14 varieties from Saudi Arabia, Egypt and Iraq [19], *7:* 12 varieties from Tunisia [18]

**Table 2:** Mineral contents of date fruit edible part and seeds (mg/100 g)

	Date fruit (edible part)		Date seed	
	1	2	3	4
Potassium	823 ± 13.1–863 ± 0.88 [8]	289.6 ± 0.8–512 ± 0.6	238–289	204.33 ± 2.6–300 ± 1.15
Phosphorus	101 ± 0.54–104 ± 0.24 [8]	12 ± 0.1–27 ± 0.01	58–70	68.33 ± 0.33–124 ± 0.58
Magnesium	41.6 ± 0.29–44.1 ± 0.97 [8]	56 ± 0.03–150 ± 0.07	48	-
Calcium	47.7 ± 0.22–63 ± 1.0 [8]	123 ± 0.4–187 ± 0.5	26–34	-
Sodium	10.1 ± 1.6–10.2 ± 0.33 [8]	4.9 ± 0.01–8.9 ± 0.02	9.573 ± 0.056–10.37 ± 0.081	10.61 ± 0.1–16.7 ± 0.6
Iron	2.0 ± 0.21–2.5 ± 0.1 [8]	-	1.763 ± 0.027–1.887 ± 0.088	-
Zinc	2.433 ± 0.03–2.467 ± 0.053 [16]	-	1.177 ± 0.046–1.363 ± 0.044	-
Cupper	2.167 ± 0.053–2.167 ± 0.107 [16]	-	1.04 ± 0.009–1.123 ± 0.023	-
Manganese	1.083 ± 0.071–1.130 ± 0.018 [16]	-	0.273 ± 0.019–0.353 ± 0.035	-

Values in most studies (mean ± S.D)

*1:* Two varieties (Deglet Nour and Allig), Tunisia [8, 16], *2:* 10 varieties from Saudi Arabia [20], *3:* Two varieties (Deglet Nour and Allig), Tunisia [16], *4:* 12 varieties from Tunisia [18]

**Table 3:** Major fatty acids in date fruit edible part and seeds as % of total fatty acids

	Date fruit edible part	Date seeds	
	1	2	3
Lauric (C12:0)	3.05 ± 0.06	17.39 ± 0.48	14.2 ± 3.4
Myristic (C14:0)	-	-	11.7 ± 1.1
Palmitic (C16:0)	20.55 ± 0.64	10.2 ± 0.43	11.8 ± 0.6
Oleic (C18:1)	23.35 ± 0.56	47.66 ± 0.67	47.0 ± 2.9
Linoleic (18:2)	32.77 ± 0.59	10.54 ± 0.28	8.6 ± 1.4
Linolenic (C18:3)	9.19 ± 0.03	0.46 ± 0.07	-

Values (mean ± S.D)

*1:* Khalti, Tunisia [17], *2:* Khalti, Tunisia [17], *3:* 14 varieties from Saudi Arabia, Egypt, and Iraq [19]

Taroone which is obtained from the inflorescence sheath of DP that is also named DP spathe distil contains proteins, fats, sugars, amino acids, and vitamins. Spathe is an envelope-like structure that encloses male and female date palm flowers (inflorescence) within it before DP pollination [24, 25]. During pollination process, the spathe is split open to expose the mature flowers for pollination. DP spathe is considered one of major DP waste products.

## Bioactive Constituents in Date Palm

DP seeds from different date varieties were reported to contain cinnamic acid and its derivatives [26–29]. Cinnamic acid and its derivatives have promising broad-spectrum biological activities including antioxidant and antimicrobial [30]. Also, coumarins of more than 1300 compounds have been identified in the seeds, roots, and leaves that were shown to have antioxidant, anti-inflammatory, antimicrobial, and anticancer activity [31–35]. Accordingly, DP seeds were reported to contain 4, 6-dimethyl-3-(4-methoxyphenyl) coumarin as 14.73%, 4-methylcinnamic acid as 11.44%, 6-hydroxy-7-methoxycoumarin as 8.71%, and 7-allyloxy-4-methylcoumarin as 2.20% of both antioxidant and immune-stimulant activity [36]. From the phenolic acids that present in the seeds were protocatechuic (7.9 mg/100 g) and caffeoylshikimic (28.3 mg/100 g). The flavonoids that present in the seeds were apigenin derivatives (0.5 mg/100 g), quercetin derivatives (3.4 mg/100 g), proanthocyanidins dimer (55.8 mg/100 g), proanthocyanidins trimer (61.3 mg/100 g), and epicatechin (18.8 mg/100 g) [37]. Analysis of hydroalcoholic leaf extract showed the presence of ten phenolic compounds [38].

**Fig. 1:**
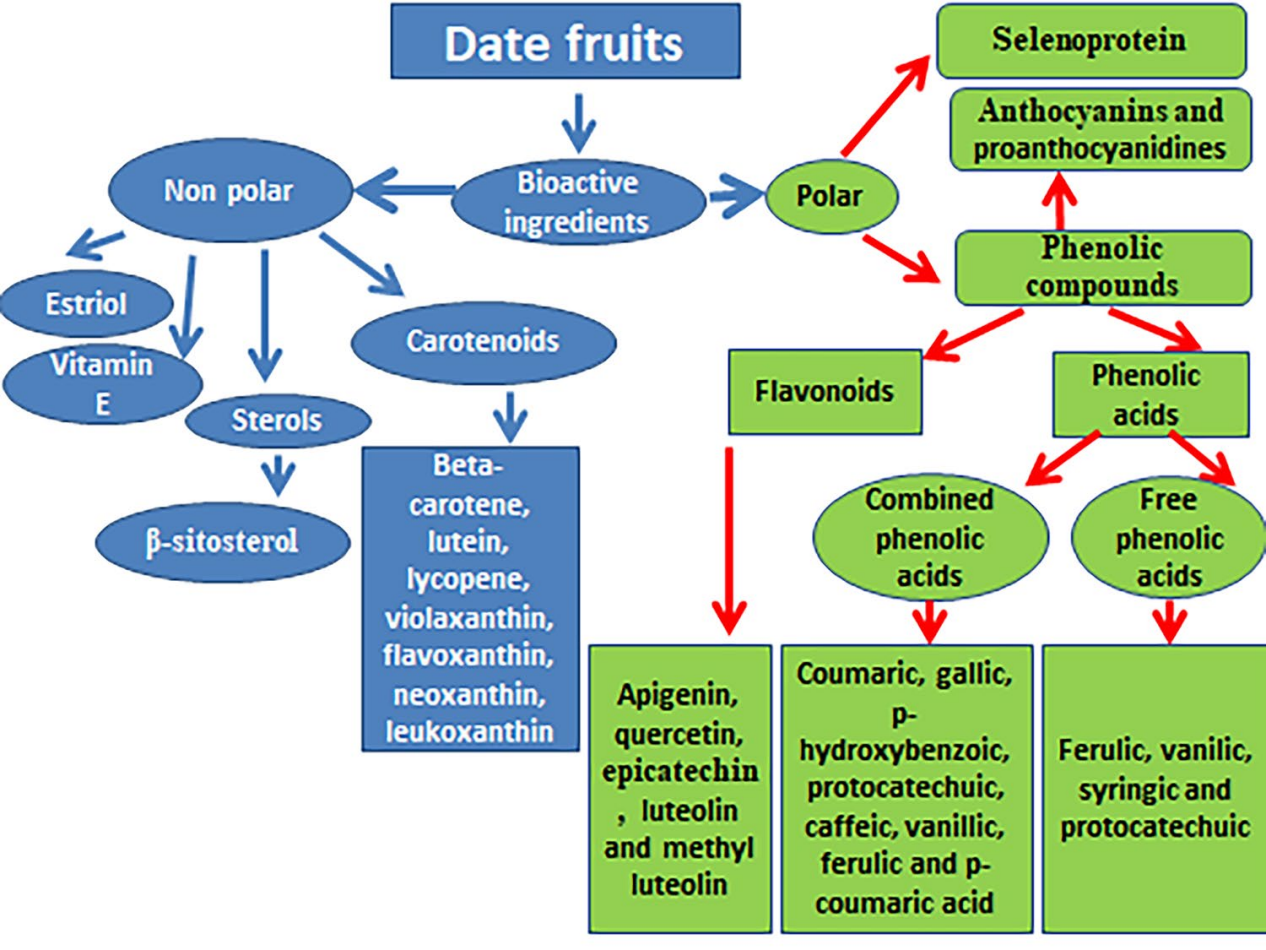
Bioactive constituents of date fruits

Date fruit is rich in phenolic compounds, carotenoids (beta-carotene, lutein, lycopene, violaxanthin, flavoxanthin, neoxanthin, leukoxanthin), and sterols [37, 39, 40•]. Carotenoids were present as zeaxanthin (10.8 µg/Kg), lycopene (19.5 µg/Kg), β-cryptoxanthin (20.4 µg/Kg), lutein (1599 µg/Kg), and β-carotene (1342 µg/Kg) in Khalas variety from United Arab Emirates [14]. It was also reported that the fruit is rich in phenolic compounds and flavonoids that possess both antioxidant and free radical scavenging activity. The free phenolic acids that present in the fruit are ferulic, vanillic, syringic, and protocatechuic, while the combined phenolic acids are symbolized by coumaric, gallic, p-hydroxybenzoic, protocatechuic, caffeic, vanillic, ferulic, and p-coumaric acid [41–43]. Ferulic acid is the major phenolic acid in Omani date [44]. The flavonoids that present in the fruit are apigenin, quercetin, luteolin, proanthocyanidins, and anthocyanins [37]. Figure 1 compiled the bioactive constituents of date fruits.

The phenolic acid levels in the fresh and dry fruit, respectively, were reported to be as the following: protocatechuic (2.27 and 4.94 mg/100 g), ferulic (9.62 and 11.83 mg/100 g), gallic (0.16 and 1.56 mg/100 g), vanillic (1.76 and 4.13 mg/100 g), caffeic (3.37 and 2.52 mg/100 g), syringic (2.45 and 6.06 mg/100 g), 4–hydroxybenzoic (0.16 and 0 mg/100 g), and p-coumaric (2.89 and 5.77 mg/100 g), while lignans were present as 323.6 µg/100 g fresh fruit [37].

The levels of different bioactive constituents in the fruit are exposed to significant changes after drying process. Anthocyanins only present in fresh fruit [45]. It was also reported that total phenolic contents increased significantly by 22–153% on drying the fruit according to its variety which ascribed to the increase in the phenolic acids by 64–107% on drying [41, 42]. Also the bioactive constituent levels are affected by the ripening stage of the fruit where the carotenoid level drops rapidly as the fruit ripens [45]. The contents of bioactive constituents differ according to fruit variety; as an example, Hallawi variety has higher phenolic level (619 mg pyrogallol equivalent and 1000 mg tannic acid equivalent/kg) than Medjool date (517 mg pyrogallol equivalent and 763 mg tannic equivalent/Kg) [46]. About 15 flavonoid glycosides like luteolin, quercetin, and apigenin exist in date in methylated and sulphated forms, so date is considered the only fruit that contains flavonoid sulfate [37, 46]. Four different DP fruits in Saudi Arabia showed the presence of phenolic content as 2153–2682 mg gallic acid equivalent/100 g dry fruit, while the flavonoids constitute 290–492 mg quercetin equivalent/100 g dry fruit [47].

Date palm spathe was shown to contain phenols, flavonoids, 3,4-dimethoxytoluene, 5,9-undecadien-2-one, β-caryophyllene, p-cresyl methyl ether, caryophyllene oxide, steroids, triterpene steroids, and bioactive essential oil [48–51]. Other constituents like furfural, calcium pectate, 1,2‑dimethoxy 4‑methylbenzene, camphor and coumarin derivatives, phytosterols, amino acids, and vitamins were also reported to be present in spathe [25, 52].

## Health Benefits of Date Palm

Different health promotion effects of nutraceuticals prepared from DP were reported. These health benefits were ascribed to different bioactive constituents that present in various parts of date palm including date fruits, seeds, leaves, and others.

**Fig. 2:**
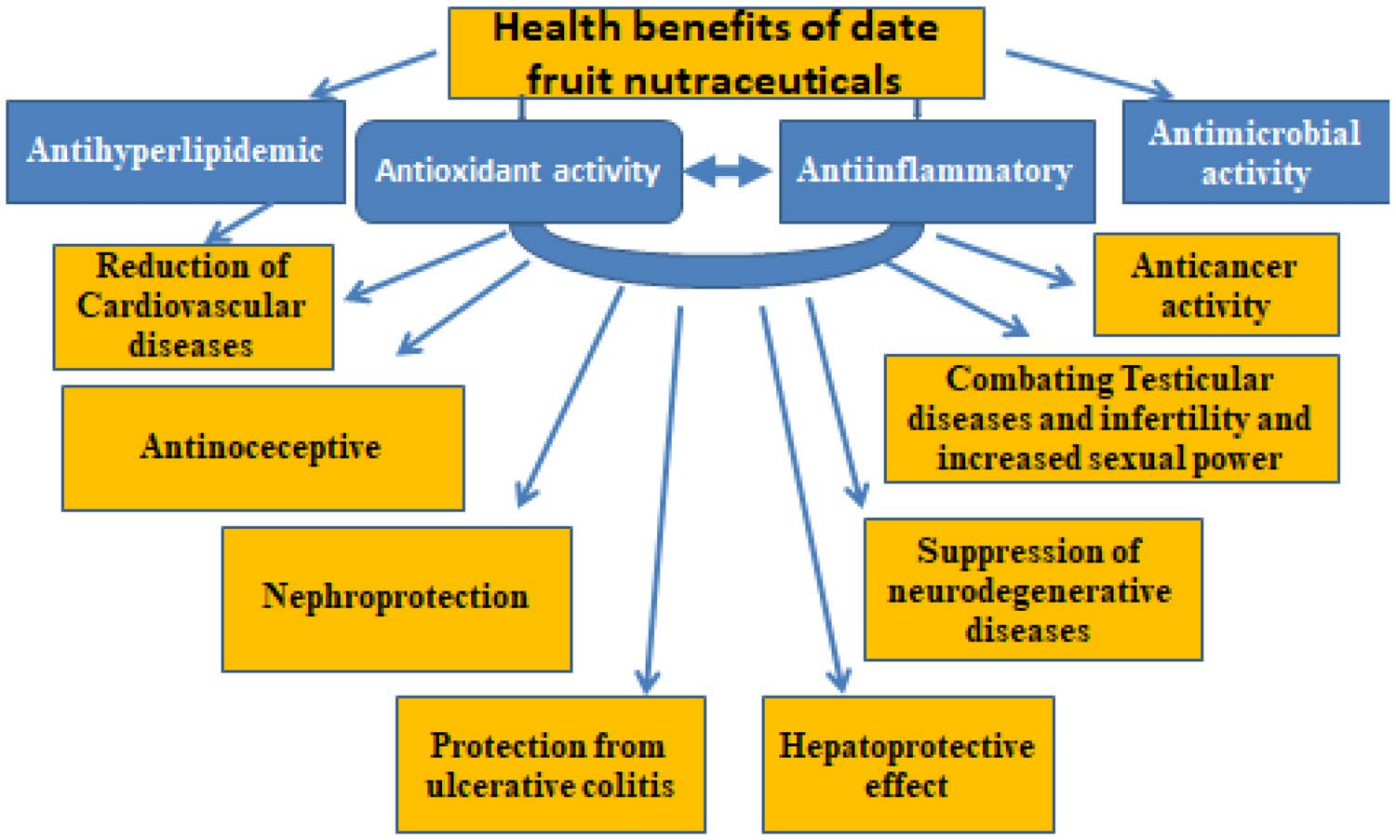
Health benefits of date fruit

Figure 2 demonstrated the different health benefits of date fruit.

### Antioxidant Activity of Date Palm

Oxidative stress is known as the most important guilt in chronic diseases. Oxidative stress results from an elevation of oxidants with concomitant reduction in antioxidants [53]. Dietary antioxidants exert their effects through elimination of reactive oxygen species and subsequently prevent the activation of the inflammatory process [54]. Date palm fruit is an excellent source of phenolics, and therefore, possesses an extremely high antioxidant capacity [55, 56]. The antioxidant activity of date palm has been attributed to the presence of selenoprotein, anthocyanins, and both phenolic and flavonoidal compounds [57]. Date palm fruit extract has a substantial neuroprotective impact against cerebral ischemia and consequent reperfusion due to its antioxidant activity [58, 59]. It was demonstrated that date fruit aqueous extract from bekraray variety native to Libya improves the plasma lipid profiles of the diet induced hypercholesterolemic rabbits through the augmentation of specific biosynthesis of antioxidant enzymes [60], and the dose levels of the extracts ranged from 125 to 1000 mg/kg and given for 10 weeks. The antioxidant enzyme activities represented by superoxide dismutase and glutathione peroxidase in addition to total antioxidant activity showed significant elevation on treatment with date fruit extract in such animal model [60].

The presence of phenolic compound either phenolic acids (ferulic and coumaric) or flavonoids (luteolin, epicatechin, quercetin, and methyl luteolin), anthocyanins, and selenoprotein impart an antioxidant activity to DP [55, 56]. In vitro antioxidant activity of the aqueous extract of date fruit is demonstrated in many studies based on its phenolic compounds with potent free radical scavenging activity [61, 62]. Petroleum ether and methanol extract of the date fruit showed in vitro antioxidant activity of 53.39 and 75.96%, respectively, calculated as percent inhibition relative to control according to beta-carotene bleaching method [63]. The antioxidant activity of the methanol extract was attributed to the presence of phenolic compounds [63, 64]. Phenolic compounds are generally good antioxidants that are known to protect cells from damage. They are known to have a preventive role against free radicals and therefore may contribute to the prevention of several age-related diseases as shown from supplementation of Mediterranean diet which is rich in phenolic compounds when tested in human [65].

Petroleum ether extract of date seed, which represents seed oil, possess antioxidant activity which was ascribed to the presence of vitamin E, β-sitosterol, and estriol reported to be present in the unsaponifiable matter of date seeds [66]. It is worthy to mention that the seed oil contains both saponifiable and unsaponifiable matter. The saponifiable matter comprises the fatty acids, while the unsaponifiable constitutes most other bioactive compounds of the oil as the aforementioned constituents. It was reported that total sterol content present as 300 to 350 mg/100 g in the varieties Allig and Deglet Nour, respectively of DP. β-sitosterol accounted for 83.31% and 78.66% of the total sterol in Deglet Nour and Allig, respectively. Alpha-tocopherol is the predominant components in both date seed oils (24.97–38.85%) [23].

Date seed protein hydrolysate was shown to inhibit oxidation in biological system and prevent low density lipoprotein-cholesterol (LDL-C) oxidation. Hydroxyl and peroxyl radicals that induced secession of DNA were effectively inhibited by the protein hydrolyzate [67•]. The incorporation of the hydrolyzates as 200 ppm in fish model system resulted in inhibition of 30% oxidation. Also, the hydrolyzates inhibited beta-carotene oxidation by 75%. The hydrolyzates (0.1 mg/ml) reduced LDL-C oxidation by 60% and inhibited hydroxyl and peroxyl radical-induced DNA scission using 6 mg/ ml and 0.1 mg/ml, respectively [67•].

Daily consumption of 100 g from Halawi date variety by healthy non-smoking subjects of 20 to 40 years old for 4 weeks produced reduction in serum oxidative stress by 33% compared to basal level. Also Halawi date inhibited serum lipid peroxidation and aryl esterase activity and therefore prevent oxidation of serum lipoprotein [46]. Diet containing 2, 4, and 6% date palm seed fed to broilers showed significant increases in serum reduced glutathione (GSH) values [36] pointing to elevating antioxidant status, and the percentage increase in GSH was ranged from 12 to 36% after feeding period of 3 weeks and from 35 to 47% after 6 weeks. Date seed oil was reported to possess antioxidant effect in ex vivo studies in normal human epidermal melanocytes and keratinocytes [68, 69]. Date seed oil produced increase in antioxidant enzymes represented by catalase, superoxide dismutase, and glutathione peroxidase by approximately 33%, 50%, and 43%, respectively, in human epidermal keratinocytes with induced hydrogen peroxide oxidative stress. Thiobarbituric acid reactive substance (as indicative of oxidative stress) was inhibited by about 42% using DP seed oil [69].

### Anti-inflammatory and Antinociceptive Activity of Date Palm

Non-steroidal anti-inflammatory drugs are used to relief pain and reduce inflammation, but unfortunately their severe side effects necessitate searching alternative natural agents. Such side effects are ranging from common to severe and are represented by nausea, vomiting, reduced appetite, gastrointestinal disturbance, dizziness, headache, and drowsiness and could be more vigorous to induce kidney failure, liver failure and ulcers, bleeding, and perforation of stomach and intestine. Pain killers or analgesics from natural sources might represent a safe approach compared to drugs. Taroone is widely used as a healer remedy for relieving tooth and joint pains and treating rheumatic diseases, as well as possessing sedative and hypnotic effects [24]. Chemically, DP spathe extract (DPS) contains proteins, fats, sugars, lignin, phenolic compounds, flavonoids, furfural, calcium pectate, 1,2‑dimethoxy 4‑methylbenzene, 3,4‑dimethoxytoluene, camphor and coumarin derivatives, phytosterols, amino acids, vitamins, moisture, and wood ash [25, 52, 70–72]. The anti‑inflammatory and analgesic effects of some of these constituents have been evaluated [73–75]. The results of a preliminary experimental trial study confirmed the analgesic effect of DPS extract in a formalin-induced pain model in male rats [76], an evidence for analgesic and anti-inflammatory effects of some spathe’s components. The fresh sheath of palm inflorescence from Bam Mazafati palm tree (*P. dactylifera*) from Iran was dried, and extracts were prepared from the dried pieces of spathe. Treatment with hydroalcoholic extract of DPS in a dose of 200 mg/kg has significant analgesic effect towards chronic pain, but not on acute pain in mice which was identical to 30 mg/kg diclofenac and 8 mg/kg morphine [77].

Camphor derivatives, which are among the DPS extract constituents, may be involved in DPS anti-inflammatory and analgesic effects, via stimulation and subsequent blockade of the sensitivity of transient receptors vanilloid subtype I channels, which are abundantly expressed in nociceptive neurons [74, 75]. Peripherally, coumarin derivatives, another DPS extract constituents, can enhance the removal of free oxygen radicals [73, 76, 78]. Following the tissue damage, similar to what occurs after intraperitoneal injection of acetic acid, the inflammatory prostaglandins E are produced in the abdominal cavity and help the process of inflammation and intensification of pain, through sensitizing the nerve endings to bradykinin, histamine, and other released transmitters [79]. Coumarin and its derivative, 7‑hydroxy coumarin, inhibit the production of such prostaglandins E through inhibition of the cyclooxygenase enzyme systems, resulting in the reduction of inflammation and pain induced by acetic acid [73]. Beta sterols that are a form of phytosterols, abundantly present in DPS extract, were also introduced as anti-inflammatory agents; however, their effect was weaker than the anti-inflammatory effects of hydrocortisone [80, 81]. A research conducted by the use of formalin, hot plate, and writing tests revealed that vitamin C, carotene, phytosterols, and calcium in the plant products were considered to induce a significant anti-inflammatory and antinociceptive effect [82]. According to the findings of another study in 2008, acetyl 2, pyronie, scopoletin (a kind of coumarin), and alpha-spinasterol (a kind of phytosterols) found in plant extracts are involved in attenuation of glutamate-induced pain in rats, through the blocking of glutamate receptors as well as by inhibiting the pro-inflammatory cytokines such as tumor necrosis factor alpha and type 1 leukotrienes [83]. In an experimental study, anticonvulsant effect of 3,4-dimethoxytoluene, as one of the major components of DPS, was investigated in four different convulsing models in rodents, and it was proposed that the anticonvulsant effects could be due to increasing the gamma-amino butyric acid levels in the brain, which inhibits the construction of nitric oxide enzymes, N-methyl-D-aspartate glutamate receptors, or by suppressing the brain noradrenergic pathways [84]. According to the suggested effects for different DPS extract constituents, the analgesic effect of dates spathe extract may be through the central pathways involved in acetic acid-induced pain modulation or via antioxidant effect of sterol and flavonoids constituents of the extract [58] which inhibit the production of inflammation-inducing factors [52, 71]. Furthermore, structural changes in the central nervous system such as increasing the number and responsiveness of alpha-2-adrenergic receptors in the spinal cord may be effective in modulating pain sensation by the extract [85].

Methanol and water extracts of date fruit Egyptian variety (Zaghlool) showed to inhibit the inflammation induced by Freund’s complete adjuvant by 67.8% and 61.3%, respectively, in rats. Methanol extract of the seed of the same variety reduced inflammation by only 35.5%. This study reported improvement of the biomarkers in adjuvant arthritis that simulate rheumatoid arthritis in human pointing to the possible health benefits of date fruit in such disease [86]. Date fruit extract when incorporated into date jam produced a functional food with anti-inflammatory and anti-arthritic effect in rat adjuvant arthritis model when the functional food was mixed as 23% of diet and fed for 4 weeks [87]. Date palm seeds anti-inflammatory activity was ascribed to its phenolic content [64]. The in vitro anti-inflammatory study of Taleb et al. [40•] confirmed the aforementioned in vivo effect.

### Antihyperlipidemic, Reduction of Cardiovascular Risk and Hepatoprotective Activity of Date Palm

Consumption of high sugar-high saturated fat diet is accompanied by obesity, hypercholesterolemia, insulin resistance, and fatty liver diseases that could certainly lead to cardiovascular diseases [88–91] which are considered the leading cause of death worldwide. Aseel date variety was reported to have hepatoprotective and antihyperlipidemic effect in rats fed on high sugar-high saturated fat diet. Aseel variety suspension (300 mg/Kg) produced significant reduction in triglycerides by 56%, total cholesterol (T-C) by 25%, LDL-C by 68%, very low density lipoprotein cholesterol by 55%, T-C/high density lipoprotein cholesterol (HDL-C) by 24%, and LDL-C/HDL-C ratio by 69% in such rats without affecting liver enzymes. Increasing the dose to 600 mg/Kg could reduce alkaline phosphatase pointing to improving liver function [92]. Date seed was shown to have antihyperlipidemic effect due to fibers’ content where date seed fibers at 1.5% concentration significantly reduced plasma concentration in rats [93]. Date fruit extract exhibited hepatoprotective activity which might be ascribed to phenolic compounds [94]. Treatment by the fruit extract restored the liver damage induced by dimethoate in rat through inhibition of hepatic lipid peroxidation, amelioration of superoxide dismutase, glutathione peroxidase, and catalase activity, along with improvement of histopathological changes [94].

Coumarin derivatives in DPS extract was reported to inhibit lipid synthesis in the body [73]; therefore, Taroone is widely used for lowering blood lipids [24]. The vasodilator, antioxidant, and anti-inflammatory activity of coumarin are among the proposed mechanism for lipid lowering and cardioprotective effect [73, 95].

### Potential Combating Testicular Diseases and Infertility and Increased Sexual Power by Date Palm

Testicular torsion is a genitourinary emergency that requires an immediate surgical correction. This condition causes ischemic reperfusion which damages the testicular tissue and affects the quality of sperm. It usually happens following the rotation of testis around the axis of the spermatic cord, and it is frequently observed in newborns, children, and adolescents [96]. Necrosis of germinal cells induced by long-time torsion might lead to infertility [97, 98]. Blood flow of the testicular tissue subsequent to the reperfusion exposes the tissue to the produced reactive oxygen species (ROS) and consequently an ischemia/reperfusion (I/R) injury [56, 99]. Oxidative stress is considered to be the main blamed in I/R injury [100–102]. Pretreatment with antioxidants can protect the testis against ROS insult [55, 103].

The antioxidant activity of water extract of the edible portion of date fruit was studied by Jahromi et al. [104] in experimental model of testicular torsion/detorsion (T/D) in which testicular ischemia was induced in rats. Before induction of testicular ischemia, rats were treated by the extracts of the edible fruit of date palm as 500 mg/kg orally. In the T/D group, serum malondialdehyde and oxidative stress were elevated significantly. These parameters were reduced in the group given date palm extract during T/D. Total antioxidant status was reduced in T/D group and increased when treated by date fruit extract. Hence, the testicular oxidative damage induced by T/D could be prevented by DP extract. Histopathological examination of testes revealed injury in T/D group; however, DP-treated group demonstrated significant improvement in both ipsilateral and contralateral testicular tissue. The significant effect on fertility and spermatogenesis induced by testicular ischemia/reperfusion injury [55, 56] were greatly improved by date palm extract compared to T/D group. This finding indicates that DP extract administration rescues spermatogenesis damage and may also prevent testicular ischemia/reperfusion injury-induced infertility.

In Folk medicine of some countries, DP pollens are used for treating infertility. Sperm count and motility, the ratio of epididymis or testis to body weight, and estradiol level were elevated on treating rats with date palm pollens (120 and 240 mg/Kg). Treatment by a dose of 120 mg/kg produced an increase in both luteinizing hormone and testosterone. Seminiferous tubule diameter increased in the three applied doses (120, 240, and 360 mg/kg) which indicated that date palm pollens could have the potential to improve fertility factors [105].

The induced spermatotoxicity due to nicotine exposure only recovered partially after cessation of nicotine exposure; however, administration of date palm pit powder could afford complete restoration in mice [106]. Also, Taroone was reported to strengthen the sexual power [24].

### Role of Date Palm Extracts as Anti-diabetic

Parthenocarpic fruit called Sish is produced by some date palms. Inhibition of the enzymes related to type II diabetes was reported by the ethanol extract of PD Sish. Box-Behnken design was used to optimize amylase inhibitor extracts from Sish. Extract prepared by 70% ethanol showed the presence of 13 phenolic compounds by liquid chromatography-tandem mass spectrometry and demonstrated more pronounced in vitro inhibition of alpha-glucosidase than alpha-amylase with IC50 (the concentration of the extract that reduced the enzymes by 50%) of 600 and 2500 μg/ ml, respectively. Postprandial hyperglycemia was improved in in vivo study on treatment with the extract referring to its possible utilization as antidiabetic nutraceutical [107]. Another in vitro study showed that the hydroalcohol extract of *P. dactylifera* leaf possessed similar result to the aforementioned study; however, it had an IC50 of 20 ± 1 and 30 ± 0.8 μg/ml towards inhibition of alpha-glucosidase and alpha-amylase, respectively. Inhibition of alpha-glucosidase and alpha-amylase enzymes can suppress carbohydrate digestion and delay glucose uptake with consequent reduction of blood sugar level [108]. Postprandial hyperglycemia was improved by the extract in a dose of 20 mg/kg which was superior to 50 mg/kg dose from the drug called glucor (acarbose) in diabetic mice when treated for 28 days [38].

### Date Palm as Regulator of Immunity

Immunity is defined as the capability to resist harmful organism, and it involves specific and nonspecific components. The nonspecific components act as barriers or eliminators of a wide range of pathogens irrespective of their antigenic make-up. Other components of the immune system adapt themselves to each new disease encountered and can generate pathogen-specific immunity. In other meaning, the immune system has innate and adaptive immunity. The innate system stimulates two types of innate immune responses: inflammatory responses and phagocytosis. On the other hand, the adaptive system is composed of more advanced lymphatic cells that are programmed to distinguish between “non-self” substance in the presence of “self”. The reaction to foreign substance is described as inflammation [109, 110].

Broiler fed on diet containing 2% DP seed produced a significant increase in both interlukin-2 (IL-2) and interferon gamma ((IFN- γ) pointing to enhanced immunity [36]. IFN-γ is a soluble cytokine that is the only member of type II class of interferon known as immune interferon [111]. IL-2 is a type of cytokine that regulates the activities of leukocytes, often lymphocytes that are accountable for immunity [112]. Moreover, when DP fruit was extracted with hot water, the extract was shown to regulate cellular immune system due to presence of phenolic compounds and polysaccharides. The numbers of spleen IFN-γ CD_4_, IFN-γ CD_49_b, and IL-_12_CD_11_b demonstrated significant increase in mice given the date fruit extract compared to those treated with prune extract, fig extract, or extract-free control diet for 30 days. Chlorogenic acid, caffeic acid, ferulic acid, and pelargonin in date palm stimulated IFN-γ m RNA expression in Peyer’s cell culture [113]. The same study demonstrated an increment of natural killer cells, macrophages, and dendritic cells in both Peyer’s patches and spleen which indicated an improvement of immune function [113]. In another study, hens fed on diet containing 0.5% DP pollen grain improved immune system. When such hens were injected with sheep blood cells as antigen and given another booster dose after two weeks, they showed a higher antibodies titer and IgM titer after the first and second injections compared to control [114].

### Anticancer Activity of Date Palm

Date palm seed anticancer activity was ascribed to its phenolic content [64]. The anti-angiogenic activity of date fruit [40•] might explain its anticancer effect. Angiogenesis which is the new formation of blood vessels has a great role in cancer growth and spreading. There are chemical signals that enhance angiogenesis and other signals that inhibit it, and they are present in balance according to the need. Tumor cell can stimulate angiogenesis chemical signaling in both tumor cells and in the nearby normal cells to form new blood vessels. Such new vessels supply tumor cell by oxygen and nutrients to invade new tissues and to be transported to produce metastasis. Therefore, angiogenic inhibitors could slow or prevent the cancer growth and metastasis [115]. It was reported that polyphenols in date syrup possess anti-angiogenic effect via inhibiting different processes like tube formation, cell migration, invasion, and metalloproteinase activity at 60 and 600 μg/ml. The anticancer activity of date polyphenols included also anti-inflammatory activity related to reduction of vascular endothelial growth factor, the prostaglandin enzyme cyclooxygenase-2 (COX-2), and tumor necrosis factor-alpha (TNF-α) gene expression [39]. It has been reported that increased prostaglandin levels have been detected in cancer and that COX-2, the key enzyme in the production of prostaglandin, is upregulated in many malignancies and can be modulated by cytokines like TNF-α. Vascular endothelial growth factor (VEGF), a potent angiogenic factor, was shown to be correlated with COX-2 in cancer. Therefore, anti-inflammatory agents could inhibit tumor growth by blockage of COX-2 leading to suppression of eicosanoids production specially prostaglandins and might affect cell proliferation, apoptosis, immune response, and angiogenesis [116, 117]. The reported anticancer effect of date fruit [61, 118, 119] could be also attributed to immune-modulatory effect [5].

Prebiotic represented by dietary fibers might promote the growth of beneficial bacteria compared to pathogenic one and thereby improve the balance of colonic microbiota. The presence of insoluble fibers in association of phenolic compounds in date fruits may have an important role in preventing imbalance of colonic microflora leading to prevention of colorectal cancer [120]. A clinical study carried out by Eid et al. [120] showed that daily feeding of 7 dates (50 g) for 3 weeks could not ameliorate either the microbiota or the colonic short-chain fatty acids. However, this treatment produced increased bowel movements and stool frequency with reduced stool ammonia and stool water genotoxicity referring to potential reduction of colorectal cancer incidence without affecting microbiota. It is proposed that elongation of the period of date palm supplementation and/or increasing its quantity could have a positive effect on the microbiota. The hydroalcoholic extract of *P. dactylifera* leaf was also efficient in inhibiting cancer cell growth of human melanoma-derived cell line (IGR-39) at dose of 35 and 75 μg/ml [38].

### Date Palm for Suppression of Neurodegenerative Diseases

The beneficial effects of DP fruits on neurodegenerative diseases like Alzheimer’s (AD) and Parkinson’s disease were reported [121–123]. AD is a progressive neurodegenerative disorders and a leading cause of dementia, which is characterized by cognitive and memory impairment. In AD, the 1–42-amino-acid peptides, cleaved from a larger protein called β-amyloid precursor protein (APP), form an amyloid-beta (Aβ) peptide that accumulates in brain and leads to neuronal death [124]. The regular intake of date reduces the exposure to neurodegenerative disorders and enhances cognitive performance in elderly [125]. The aqueous date fruit extract has been shown to prevent neuronal circuitry against focal cerebral ischemia [59]. The amounts of phenolic, flavonoid, and antioxidant contents vary from variety to variety of date fruits. The comparative analysis of three major date palm varieties (Fardh, Rutab, and Khalas) available in Oman have shown that all the varieties had high phenolic and flavonoid contents that exhibit neuroprotection against the induced cell death in human neurons [126]. These varieties also demonstrated neuroprotective effect against the oxidative stress in a transgenic mouse model of AD [123, 126].

The long-term (15 months) dietary supplementation of date fruits attenuated the levels of Aβ peptides and adenosine triphosphate and possessed anti-inflammatory effect in mice model of AD compared to aged wild mice. The supplementation of date fruits (4% of diet) significantly decreased pro-inflammatory cytokines represented by interleukins, tumor necrosis factor alpha, and eotaxin in APPSw2576 transgenic mice of AD [121]. Date fruit supplementation also delayed senile plaques formation with the decrease in brain Aβ_1–40_ and Aβ_1–42_ contents that have been shown to be associated with the protective role of dates against AD. Long-term cerebral hypoperfusion in rats has been shown to cause a propensity towards anxiety and restlessness accompanied by deficits of spatial learning and memory. Post-occlusion treatment for 15 days with 100 and 300 mg/kg doses of methanol extract of date fruits significantly reduced the levels of malondialdehyde in brain. This extract prevented neuronal necrosis as evidenced by histopathological observations in hypoperfused brains [127].

The DP fruits have anti-inflammatory effect and protect against oxidative stress in brain that might be ascribed to their phenolic content, which clearly demonstrates the nutritional and medicinal values of this fruit [55, 56, 58, 59, 86]. Therefore, therapeutic potential of these fruits towards AD was postulated. However, the mechanisms by which date palm fruits display their antioxidant activities against the AD are poorly understood and necessitate an extensive investigation utilizing different varieties.

Supplementing the diet with 2% and 4% Omani date palm fruit could improve cognition and behavior in AD transgenic mouse model. The effects include recovering from anxiety, impairment of spatial and position learning ability, and improving motor coordination. Aβ protein was reduced significantly in the brain of mice fed on date palm-supplemented diet. The health effect was correlated to date level. Therefore, date fruit could lower the progression and delay the onset of AD through its neuroprotective effect [123]. The anxiety in AD is considered a problematic symptom that could be ameliorated by date fruit [128], an effect that might be related to antioxidant and anti-inflammatory activity. Among the abundant phenolic compounds in Omani date is ferulic acid [129–131]. Long term administration of ferulic acid (4 weeks) was shown to inhibit the negative effect of Aβ 1–42 in mice brain [132, 133]. Ferulic acid also reduced Aβ deposition in the mice brain and produce destabilization of Aβ fibrils in vitro [134–136]. Protection of neuronal cell death due to cerebral ischemia was demonstrated by intra-venous ferulic acid [137–139]. Ferulic acid promotes neuronal progenitor cell proliferation in both in vitro and in vivo studies thereby ameliorate stress-induced depression [140] and anxiety. Ferulic acid improves behavior impairment and reduces amyloidogenic metabolism by modulation of β-secretase leading to mitigation of AD-like pathology in mice [141]. Other phenolic compounds present in date palm like protocatechuic acid and caffeic acid protect against Aβ induced toxicity [142, 143]. Caffeic acid inhibits tau phosphorylation, calcium influx, and oxidative stress involved in AD [143, 144]. Supplementation of the whole date fruit could prevent oxidative stress related changes in AD model in mice [123, 126].

### Nephroprotection by Date Palm

It was reported that date palm seed aqueous extract possessed renoprotective activity in rat diabetic model through improving kidney function and reducing oxidative stress which was ascribed to the antioxidant activity and free radical scavenging activities of DP seeds active constituents [145]. Therefore, this extract could improve renal dysfunction which is one of diabetic complication. Both seed and edible portion extract of DP produced protection from gentamicin induced nephrotoxicity that was ascribed to the presence of melatonin and vitamin C and E [146].

### Protective Role of Date Palm Towards Ulcerative Colitis

Ulcerative colitis is an inflammatory bowel disease characterized by inflammation, mucosal damage, and ulcers of the colon and rectum. Different theories have been proposed for the pathogenesis of the disease from which the most acceptable is an autoimmune mechanism. Recently, a mixture of alcohol extract and petroleum ether extract of date seed from Egyptian El Barhy variety was demonstrated to possess protective effect towards ulcerative colitis model in rats in addition of prevention of iron deficiency anemia in such model [147••]. The proposed mechanism of action included antioxidant, anti-inflammatory, and immune-modulatory effects. The extracts’ mixture from the seed improved iron status through amelioration of hemoglobin, plasma iron, total iron binding capacity, percentage transferrin saturation, soluble transferrin receptor, and erythroferrone. In colon tissue, the extracts’ mixture was capable of reducing malondialdehyde and nitric oxide with elevating the reduced glutathione along with concomitant reduction of plasma alkaline phosphatase activity denoting elevation of antioxidant level, reduction of oxidative stress, and inhibition of colon damage. Regulation of immune system and anti-inflammatory activity by date palm seed extracts’ mixture were represented by reduction of colon interleukin-6 and down-regulation of the expression of colon interleukin -1β, nuclear factor kappa light chain enhancer of activated B cells (NF-kB), inducible nitric oxide synthase (iNOS), and cyclooxygenase-2 (COX2). Disease index of ulcerative colitis manifested by colon macroscopic examination, feces consistency, bloody feces, and body weight were improved on treatment with date seed extracts’ mixture. Also, histopathology of the colon was amended on administration of the seed date extracts’ mixture. The major phenolic compounds in the seeds of Egyptian Barhy variety were chlorogenic acid, *p*-hydroxybenzoic acid, catechin, and caffeic acid, while the prominent unsaturated fatty acids were oleic and linoleic [147••].

Table 4 outlined briefly the health benefits of different parts of DP in animal experiments, while Table 5 compiled other clinical studies dealing with date palm.

**Table 4:** Health benefits of different date palm parts in experimental animals

Beneficial health effects	Date palm part	Reference no
Antioxidant activity	Aqueous fruit extract	[60]
	Seed protein hydrolysate	[67•]
	Seed	[36]
Analgesic effect in chronic pain	Hydroalcoholic extract of Taroone	[77]
Anti-inflammatory effect	Methanol and water extract of the fruit	[86, 87]
	Methanol extract of seed	[86]
Antihyperlipidemic effect	Suspension of fruit	[92]
	Date seed	[93]
Hepatoprotective	Suspension of fruit	[92]
	Fruit extract	[94]
Combating testicular ischemia and infertility	Water extract of fruit	[104]
Potential improve of fertility factors	Pollen grains	[105]
Prevention of spermatotoxicity induced by smoking	Seed	[106]
Antidiabetic effect	Parthenocarpic fruit (Sish) alcohol extract	[107, 108]
Regulation of immunity	Seed powder	[36]
	Water extract of the fruit	[113]
	Pollen grain	[114]
Anticancer activity	Fruit polyphenol syrup	[39, 40•]
	Leaf hydroalcoholic extract	[38]
Suppression of neurodegenerative diseases	Aqueous fruit extract	[59]
	Date fruit	[123]
	Date fruit	[121]
	Methanol extract of the fruit	[127]
Nephroprotection	Seed aqueous extract	[145]
	Seed and fruit extract	[146]
Protection from ulcerative colitis	Mixture of alcohol and petroleum ether extract of seed	[147••]

**Table 5:** Major health benefits of date palm from human trials

Study reference no./Criteria	Aim	Subjects	Place of the study	Period of the study	Conclusion
[148] (well controlled)	Assessing hemoglobin level and bowel movement during consumption of date fruit in comparison to iron governmental supplement and two other date products	Anemic adolescent girls	Indonesia	A month	Date can be used instead of iron tablets for management of anemia. Iron supplements have negative impacts on bowel movement, while dates have positive effect
[149] (a pilot study)	Studying the management of mucositis by date palm pollen grain oral suspension during radiation and chemotherapy	Head and neck cancer patients	-	42 days	Reduction of mouth pain and oral cancer by palm pollen grain oral suspension
[150] (well controlled)	Evaluation of date palm pollen capsules on orgasm and sexual satisfaction in postmenopausal women	60 menopausal women	Iran	35 days	Date palm pollen improve orgasm but has no effect on sexual satisfaction in postmenopausal women
[151•] (A non-randomized controlled trial)	Assessing the effect of Ajwa (date fruit) intake by cancer pediatric patients on the number of infection and hospitalization due to fever, neutropenia and mortality	56 cancer pediatric patients	Jeddah, Saudi Arabia	2008–2017	Ajwa intake improved the treatment outcome
[152] (Randomized clinical trials)	The research investigate the effect of consumption of date fruit on the amount and duration of postpartum bleeding	100 nulliparous 18–35 years old women	Mashhad, Iran	Mother given date fruit100g/day for 10 days starting 2 h from delivery	Postpartum consumption of date fruit reduced the amount of bleeding
[153]	The effect of date seed powder (Deglet Noor dates) intake on antioxidant status and oxidative stress of premenopausal women	31 premenopausal women	Gununglurah Village, Banyumas, Indonesia	2 weeks as daily administration of 250 ml made by adding 250 ml boiling water to 2.5 g of seed powder	Date seed powder elevate antioxidant status and reduce oxidative stress damage in the studied subjects

## Conclusion and Prospective Researches

Long-term exposure of human body to high oxidative stress and inflammation are the main causes of chronic non-communicable diseases. The presence of antioxidant and anti-inflammatory phytochemicals and phytonutrients in different parts of DP (edible portion of fruit, seed, leaves, spathe, and pollen grains) might be the key mechanism of protective and remedial effects of the extracts prepared from such parts towards chronic diseases.

Phytochemicals and phytonutrients present in the edible portion of date fruits were reported to be phenolic acids, flavonoids, selenoproteins, anthocyanins, carotenoids, vitamin E, phytosterols, and fibers. The health benefits of edible portion of DP fruit and its extracts include antioxidant, anti-inflammatory, hepatoprotective, hypolipidemic, anticancer, and neuroprotective effects. The fruit and its extracts also reduce spermatogenesis damage, infertility, neurodegenerative diseases, and bleeding.

The DP seed contain phenolic acids like cinnamic acid, flavonoids, coumarins derivatives, vitamin E, phytosterols, and fibers. The DP seed extracts and powder were shown to possess antioxidant, anti-inflammatory, anticancer, immunoregulatory, antihyperlipidemic, and nephroprotective effects. The DP seed powder prevented spermatotoxicity induced by smoking. A mixture of alcohol and petroleum ether extracts of date seed prevents ulcerative colitis in rats.

The DP leaf extracts contain phenolic compounds and coumarins’ derivatives of antioxidant, anti-inflammatory, antidiabetic, anticancer, and immunoregulatory activities. Taroone extract contains camphor, coumarins’ derivatives, phytosterols, calcium, flavonoids, and 3,4-dimethoxytoluene. Taroone has anti-inflammatory, analgesic, anticonvulsant, antioxidant antihyperlipidemic, vasodilatory and cardioprotective effects and strengthens sexual power. To the best of our knowledge, Taroone is not utilized well as source of nutraceuticals and nutrients. Very scarce literatures are only available concerning investigating the nutritional value and health benefits of Taroone; therefore, future studies are recommended on it.

Date palm pollens have the potential to improve fertility factors and reduce mouth pain and oral cancer. Ethanol extract of parthenocarpic fruit called Sish produced by some DP possess anti-diabetic effect due to presence of specific phenolic compounds.

As could be noticed, the different health claims and phytochemicals in the various parts of DP were investigated, but the flesh part of the fruits and the seeds were extensively studied more than the other parts. Therefore, it is important to implement comprehensive studies on the different parts of date palm. Also, experimental animal studies showed diverse health benefits of date palm which must be confirmed by clinical studies. Few well-controlled human trials utilizing DP are present, but it is required to be extended to include all the health claims verified in experimental animal studies. Although various DP varieties were studied, there are still different varieties of DP that present in different regions of the world that are not thoroughly investigated. Therefore, wide spread prospective studies are required to set a comparative investigation of bioactive constituents, nutrients contents, and health benefits of the different varieties.

From the aforementioned studies, it can be concluded that date palm is a precious source of bioactive ingredients that could be utilized for development of nutraceuticals for protection and treatment of different chronic diseases. However, further prospective clinical studies are required to confirm such health benefits and the underlying mechanisms of action. The effect of DP fruits, seeds, and other parts on colonic microbiota requires extensive studies in future researches since it might be involved in the mechanism of action of DP for prevention of different chronic diseases. In addition more nutritional and phytochemical studies are required to be implemented on different parts than fruit and seeds.
